# Application of XGBoost model for in-situ water saturation determination in Canadian oil-sands by LF-NMR and density data

**DOI:** 10.1038/s41598-022-17886-6

**Published:** 2022-08-17

**Authors:** Strahinja Markovic, Jonathan L. Bryan, Reza Rezaee, Aman Turakhanov, Alexey Cheremisin, Apostolos Kantzas, Dmitry Koroteev

**Affiliations:** 1 Centre for Petroleum Science and Engineering, Skolkovo Institute of Science and Technology, Sikorsky Street 11, Moscow, Russian Federation 121205; 2 Curtin University, Kent Street, Perth, Bentley, WA 6845 Australia; 3 University of Calgary, Calgary, AB Canada; 4 PERM Inc., Calgary, AB Canada

**Keywords:** Crude oil, Statistics, Characterization and analytical techniques, Geophysics

## Abstract

Water saturation determination is among the most challenging tasks in petrophysical well-logging, which directly impacts the decision-making process in hydrocarbon exploration and production. Low-field nuclear magnetic resonance (LF-NMR) measurements can provide reliable evaluation. However, quantification of oil and water volumes is problematic when their NMR signals are not distinct. To overcome this, we developed two machine learning frameworks for predicting relative water content in oil-sand samples using LF-NMR spin–spin (T_2_) relaxation and bulk density data to derive a model based on Extreme Gradient Boosting. The first one facilitates feature engineering based on empirical knowledge from the T_2_ relaxation distribution analysis domain and mutual information feature extraction technique, while the second model considers whole samples’ NMR T_2_-relaxation distribution. The NMR T_2_ distributions were obtained for 82 Canadian oil-sands samples at ambient and reservoir temperatures (164 data points). The true water content was determined by Dean-Stark extraction. The statistical scores confirm the strong generalization ability of the feature engineering LF-NMR model in predicting relative water content by Dean-Stark—root-mean-square error of 0.67% and mean-absolute error of 0.53% (R^2^ = 0.90). Results indicate that this approach can be extended for the improved in-situ water saturation evaluation by LF-NMR and bulk density measurements.

## Introduction

The ever-growing energy demand and volatile oil prices are driving the development of new technologies to optimize hydrocarbon production and increase recoverable reserves from conventional and unconventional deposits. To better understand their amount, petrophysical surveying is regularly employed for quantifying the in-situ oil and water saturations in reservoirs. Determining water saturation is one of the most challenging tasks involving many techniques that typically produce significantly different estimates of original oil in place (OOIP)^1^. In unconventional reservoirs, the accuracy of these estimations becomes even more important as it directly impacts the development of enhanced oil recovery (EOR) strategy. The same applies to oil-sands bitumen deposits, which are estimated to account for 30% of total world reserves^2^.

Conventionally, in-situ saturations of fluids are determined through resistivity logging. However, due to the mixed or low salinity of formation water that may be present in shallower parts of the oil-sands reservoir, the water saturation volumes can be over or underestimated^1^. The low-field nuclear magnetic resonance (LF-NMR) logging tools have proved to be a valuable alternative since the measurements are non-invasive and independent of lithology. In the past 20 years, NMR measurements were used for water–oil emulsions characterization^3^ and in recent years for the in-situ fluid saturation determination^4,5^. Although the application of NMR measurements have been demonstrated to be successful for emulsion characterization, they are limited to emulsions with high water content (> 10 wt%), and it usually requires reference measurements of oil and water NMR amplitudes necessary for obtaining additional parameters such as relative hydrogen index^3,6^. Furthermore, the application of NMR emulsion models in well-logging is even more problematic due to the alteration of NMR relaxation behavior of fluids confined in the reservoir pore space. One of the well-known approaches that had considerable success in addressing this effect is based on NMR spin–spin relaxation (T_2_) distribution peak deconvolution^7^. The concept behind this approach is that viscous bitumen relaxes faster in an NMR distribution even than surface bound water, so early T_2_ signals are attributed to in-situ bitumen, and later T_2_ signals correspond to the water saturation in the rock. Unfortunately, in cases when the NMR T_2_ distribution contain signals from bitumen, water films on grains surrounded by bitumen or heavy oils, and fast-relaxing clay bound water, a significant overlap of the signals occurs, making their separation challenging. Additionally, this approach requires not only the determination of water and oil NMR amplitudes but also the independent measurement of their volume or mass. Alternative methods involve 2D LF-NMR measurements, where instead of using one NMR parameter (i.e. T_2_ relaxation), additional parameters are employed (i.e. T_1_ relaxation or diffusion) to obtain so-called 2D NMR maps^8–10^ which can theoretically help to separate these overlapping bitumen and water signals. Application of 2D maps showed considerable success in fluid saturation evaluation, compared to 1D T_2_ relaxation distribution analysis, since T_1_ relaxation or diffusion of reservoir fluids can be sufficiently different, thus enabling relatively simple separation of their signals. However, 2D NMR is slower and more expensive to run, and there can still be instances where these signals are not distinct, in which case estimation of fluid types and fluid volumes can be challenging and require advanced analysis involving blind-source signal separation (BSS), clustering algorithms, and a certain degree of knowledge in 2D NMR maps interpretation^5^.

In the past few years, a noticeable surge of machine learning applications in the petrophysical well-logging has been seen, ranging from improved interpretation of logs^11^ and forecasting of stress in horizontal wells^12^ to the utilization of neural networks to synthesize the artificial NMR T_2_ logs^13^. In this study, two machine learning techniques are employed to improve the water content determination in oil-sands, using as inputs the T_2_ relaxation and bulk density data, along with Extreme Gradient Boosting (XGBoost) algorithm. The first modeling approach is based on a feature engineering process that reduces the number of inputs while maximizing model generalization capacity. This was achieved by deriving new features using empirical knowledge from the T_2_ distribution analysis domain and a feature extraction technique based on information theory. In contrast, the second approach considers as an input the whole NMR T_2_ distribution of the sample, aiming to preserve all available information originating from fluids residing in the sample pore space. The dataset comprised 82 oil-sands core samples recovered from northern Alberta in Canada. Water content percentage relative to of the total mass of the sample was determined by Dean-Stark extraction (%DS-w). The model training and prediction test scores of the models were evaluated using three statistical metrics and a leave-one-out cross-validation (LOOCV). These scores were compared with water content predictions based on the previously published deconvolution approach^7^.

## Theory

### LF-NMR measurements for water saturation determination

LF-NMR logging tools are measuring the response of hydrogen protons in fluids rich in hydrogen, such as oils, bitumen and water. As these tools are primarily sensitive to liquids, the response from solids (reservoir rock) remains invisible in the LF-NMR T_2_-relaxation distribution. As the physicochemical properties of these liquids vary, their relaxation will vary accordingly, thus allowing inference of their properties. LF-NMR measurements are performed in two steps: first, the NMR probe introduces the external magnetic field which polarizes the H protons (T_1_-relaxation); and second, the subsequent series of short radio-frequency pulses are applied in order to produce the signal decay curve, which represents the relaxation of H protons returning to the previous state (T_2_-relaxation). After mathematical inversion of decay curves, the T_2_ relaxation can be represented time-domain. Three main processes comprise the total T_2_-relaxation including bulk relaxation, surface relaxation, and diffusion relaxation due to the gradient in a magnetic field. In this work, the benchtop LF-NMR relaxometer was used in which the gradient is absent, thus the diffusion term can be neglected.

1 $$\frac{1}{{T_{2} }} = \frac{1}{{T_{2B} }} + \frac{1}{{T_{2S} }}$$

2 $$\frac{1}{{T_{2s} }} = \rho_{2} \left( \frac{S}{V} \right)$$

*T*_2*B*_ represents the relaxation occurring in bulk fluids or fluids in large pores, *T*_2*S*_ quantifies the relaxation of fluids in smaller pores. Also, *ρ*_2_ is *T*_2_ surface relaxivity, *S/V* is a ratio of the fluid volume and surface of the pore. Each of these mechanisms will contribute to the total relaxation in varied proportions depending on reservoir rock properties and physicochemical properties of the fluids such as rock wettability, pore size and pore surface area, fluid viscosity and chemical composition.

**Figure 1:**
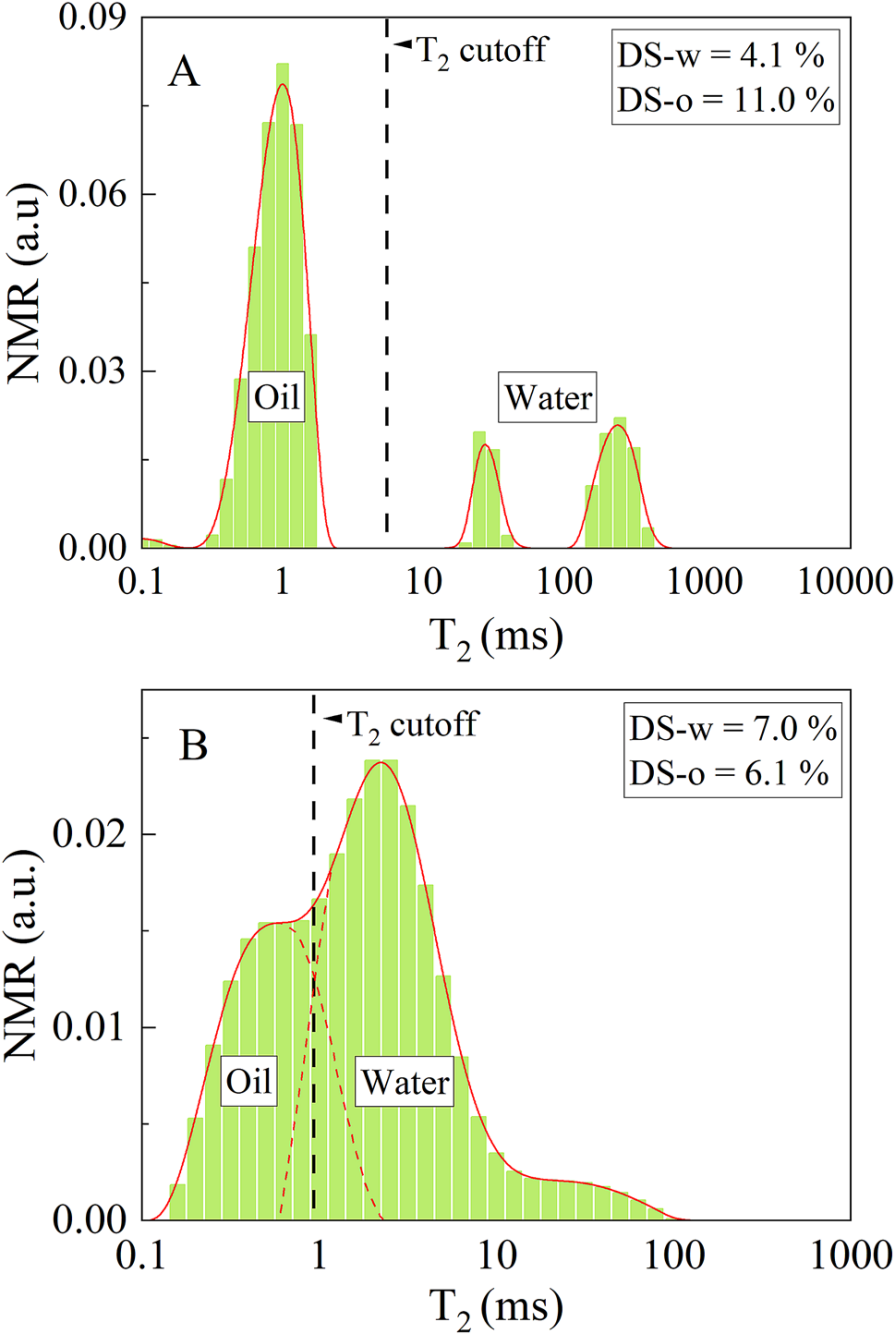
Representative NMR T_2_ distributions of two oil-sand samples. (**A**) An example of distinct oil and water signals where a simple cutoff method can be used for oil–water separation. (**B**) An example of NMR T_2_ distribution with overlapped oil and water signals where deconvolution with T_2_ cutoff cannot provide a satisfactory solution. Black vertical dashed lines present potential cutoff times. DS-w and DS-o are percentages of water and oil by Dean-Stark, respectively, relative to solids.

Assuming that the oil-sands are largely water-wet, water will generally be found in the corners of connected sand grains and potentially also as a thin film over the grain surface. The principal relaxation mechanism of hydrogen protons in high viscosity oils and bitumen would be bulk relaxation, while water would be under the strong influence of surface relaxation, and with bulk relaxation playing a smaller role in the water T_2_ values. Bulk relaxation and surface relaxation times of water and oils are unique for the most part, that is, the oil molecules relax generally quicker relative to the water molecules. When the NMR T_2_ distribution contains discrete responses of oil and water (Fig. 1A), a simple cutoff method can be applied to separate their amplitudes and quantify their volumes. However, in fines and clays, where pores are smaller, the water protons relax faster due to the surface relaxation at the water–rock interface, thus generating the signal in the fast-relaxing part of distribution where it can overlap with the signal originating from heavy oil and bitumen (Fig. 1B). In addition to that, the diffusion coupling effect may further decrease the interpretability of the oil and water signals. This effect occurs in saturated and connected micro- and macropores when water is in diffusional exchange, causing the change in the relationship between T_2_ relaxation and pore size distribution^14^. In strong diffusive-coupling conditions, macro- and micro pore water signals will merge into a single peak, rendering the single T_2_ cutoff and deconvolution approach inaccurate^15^.

In these cases, signal deconvolution can be challenging, requiring the application of a more sophisticated separation method.

Among other factors influencing the T_2_ distribution of saturated core samples, the reports in the literature showed that the relative increase or decrease of fluid saturation produces systematic shifting in sample T_2_ distribution which can be modelled and used for monitoring water saturation change^16^. As surface relaxation and diffusive coupling play a vital role in NMR petrophysical studies of porous media, the study findings also confirmed the change of dominating relaxation mechanisms with sample saturation. Another factor influencing water fraction quantification is the clay content and type^17^. Studies show that different types of clays such as illite, smectite, and kaolinite can be distinguished from NMR measurements, particularly by 2D mapping. As these clay minerals adsorb and bond variable amounts of water molecules, knowledge of their relative fraction could contribute to the more precise differentiation of bound and producible fluids from NMR measurements.

### XGBoost principles

XGBoost stands for Extreme Gradient Boosting (XGB), and it presents an implementation of the gradient boosting decision trees^18^. The main principle of gradient boosting is to utilize the individual weak learner, such as decision tree, and in a stage-wise manner, add iteratively new trees, to minimize further the objective function. This process continues for the specified number of boosting iterations, after which the prediction model is obtained in a final form. To achieve this effectively, the algorithm uses gradient descent to minimize the objective function, by finding the direction of the “steepest” descent. XGBoost on the other hand employs a number of improvements resulting in better overall generalization and computational speed. Some of the key advantages are the use of second-order gradients which contribute to a better understanding of the direction of the loss function minimum, and enhanced regularization techniques such as lasso regression (L1) and ridge regression (L2) which reduce the model complexity and overfitting. The XGBoost model can be expressed as:

3 $$\hat{y}_{i} = \sum\nolimits_{k = 1}^{K} {f_{k} \left( {x_{i} } \right)}$$

where *ŷ*_*i*_ is predicted Dean-Stark water content (%DS-w), *x*_*i*_ is a vector of input features and *f*_*k*_ is a tree at the *k*-th instance. A new tree *f*_*t*_ is added iteratively by minimizing the objective function as:

4 $$f_{t} = \sum\nolimits_{i = 1}^{n} {L\left( {y_{i} , y_{i}^{{\left( {t - 1} \right)}} + f_{t} \left( {x_{i} } \right)} \right) + \Omega \left( {f_{t} } \right)}$$

where *L* presents the specified loss function, y_i_ is observed %DS-w in a sample, *ŷ*_*i*_^*(t-1)*^ + *f*_*t*_*(x*_*i*_*)* is the predicted %DS-w at the t−1 iteration, and *Ω* is a regularization term, or a penalty function. Regularization term can be denoted as:

5 $$\Omega \left( {f_{t} } \right) = \gamma T + \frac{1}{2}\lambda \sum\nolimits_{j = 1}^{T} {w_{j}^{2} }$$

where *γ* is L1 and *λ* is L2 regularization parameters, *T* is the number of leaf nodes in a tree, and *w*_*j*_ are the weights of leaves. The addition of new trees *f*_*t*_ is performed in a stage-wise manner such that the loss between the prediction and observation is minimized, with respect to the regularization term Ω to prevent the overfitting and gauge the model complexity. Smaller values of *Ω* enable the better generalization of a tree. The detailed mathematical description of the XGBoost algorithm and additional tuning and regularization parameters is available elsewhere^18^.

## Methodology

### Experimental procedure and data preprocessing

Oil-sand samples were collected in northern Alberta in Canada, from a single delineation well. Two sets of 82 whole core samples were recovered. The first set was used for laboratory LF-NMR measurements, and a second set represented sister samples used in Dean-Stark extraction for determining the relative fraction of water, oil, and solids. Samples for NMR experiments were stored in glass vials, and measured using a Corespec 1000™ benchtop LF-NMR relaxometer, at reservoir temperature (6 °C) and ambient temperature (25 °C). The Carr-Purcell-Meiboom-Gill (CPMG) pulse sequence was used for obtaining T_2_-relaxation distribution. The CPMG parameters were predetermined after a series of test NMR experiments on different oil-sand samples. There were two aspects which had to be taken into account. The first was to tune the CPMG parameters to detect the fast relaxing heavy oil and clay-bound water signals. This was achieved by setting the shortest echo time TE that the equipment allowed (0.2 ms). The second aspect was achieving a lower signal-to-noise ratio (SNR) to simulate the well-logging in-situ NMR tool output by reducing the number of trains, which in turn resulted in a noisier signal. After trial rounds of measurements, the following parameters were found to produce optimal T_2_ distribution and SNR (Table 1).

**Table 1:** Optimal CPMG pulse sequence parameters for detection of fast relaxing clay-bound water and heavy oil signals.

CPMG pulse parameters	Values
Echo time, TE (ms)	0.2
Number of pulses, Np	5000
Wait time/post train delay (ms)	6500
Number of trains, Nt	10

For the dataset, the range of SNR varied from 5 to 56 with an average of 23. The ExpFit in-house software for multi-exponential analysis of the NMR signal was used. The representation of the signal after Inverse Laplace Transform (ILT) was obtained using Tikhonov regularization^19^. The practice has shown that the regularization parameter helps avoid oscillations in solution associated with noise and provides smooth T_2_ distributions^20^. The regularization parameter can be determined by direct and indirect methods such as Butler-Reed-Dawson, L-curve, or generalized cross-validation^20^. In the case of oil-sands, after initial analysis, the regularization parameter was determined directly and α = 0.05 was found to provide the most stable solution for most of the samples. The density values of these samples were measured beforehand by X-ray Computed Tomography (X-ray CT) using GE 9800 CT scanner, as a substitute for the density logging data.

**Figure 2:**
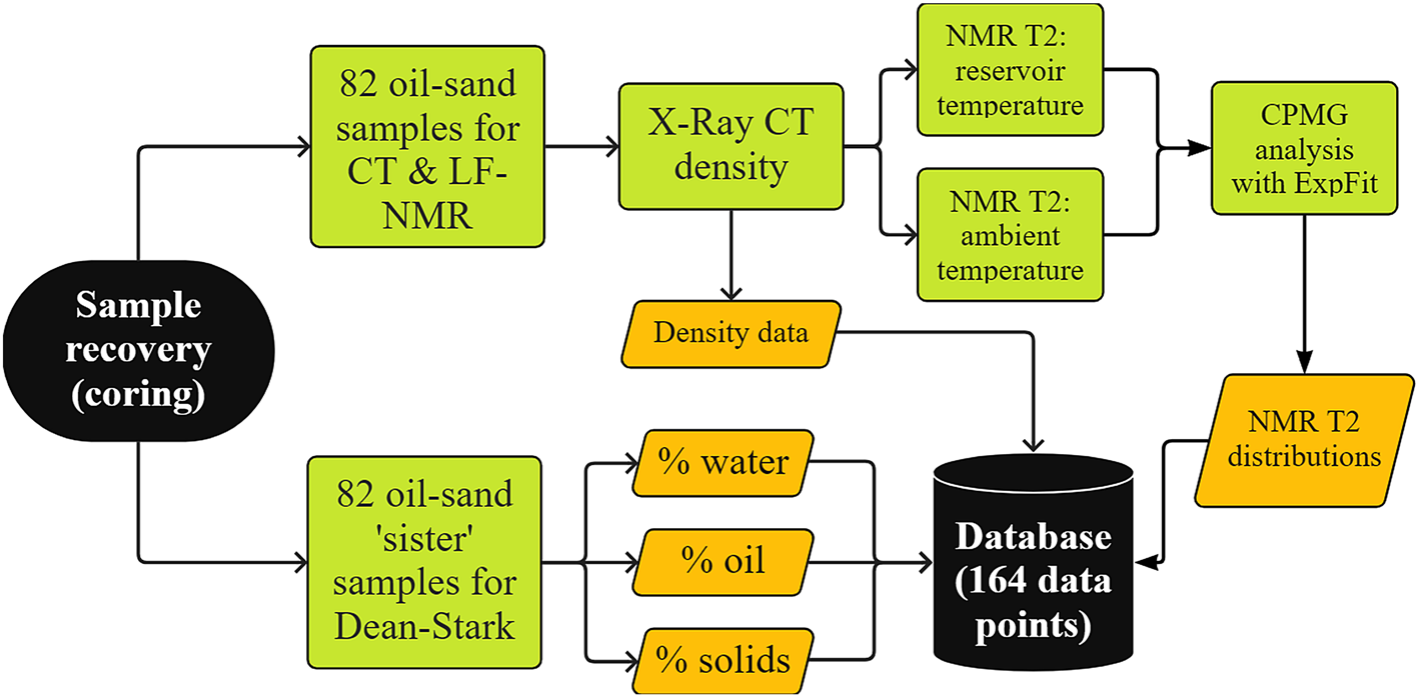
Flowchart representing the experimental program for oil-sands samples, by X-ray CT, LF-NMR T_2_ measurements and Dean-Stark extraction.

The experimental program for Dean-Stark extraction, LF-NMR measurements, and X-ray CT density measurements is illustrated in the flowchart (Fig. 2).

The final size of the dataset comprised 164 data points—82 T_2_ distributions at ambient temperature, and 82 T_2_ distributions at reservoir temperature, with corresponding density data and Dean-Stark sample composition. To compare the performance of machine learning models with the well-known peak deconvolution approach, the prediction of water content by LF-NMR measurements was also performed using the T_2_ cutoff approach developed by Bryan et al.^7^.

The data processing and model training was performed in Python 3.9 environment, while figures were produced using OriginPro 2019b software. For XGBoost model development and training, the dataset was randomly split with data shuffling into a training set and a test set in 0.25:0.75 proportion respectively. To ensure the reproducible split of the data, a random split seed was fixed to *random_state* = *2*. The XGBoost models were optimized using Bayesian Optimization (BO), while the training quality was evaluated by leave-one-out cross-validation (LOOCV). The forecasting performance of the models was evaluated using three error metrics, and residual distribution analysis. These steps will be discussed in detail in the following sections.

### XGBoost model based on feature engineering (XGB-FE)

**Figure 3:**
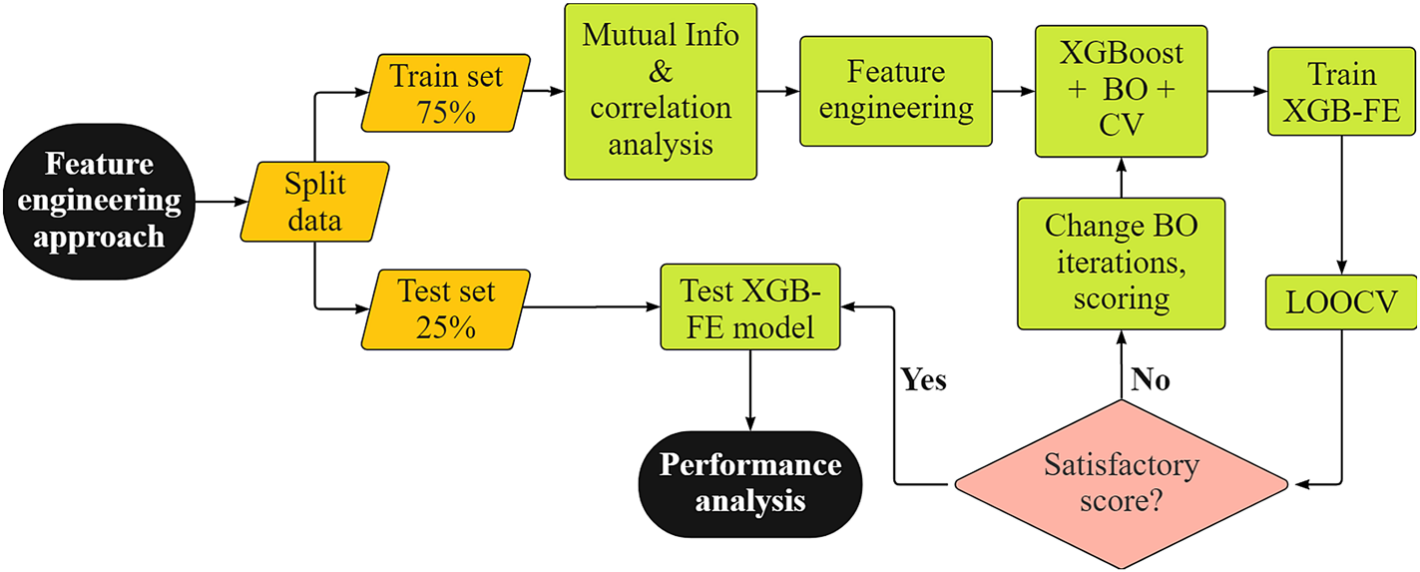
Flowchart for XGB-FE model development.

Feature engineering (FE) is a process in a part of a machine learning pipeline where domain knowledge is utilized to extract the most relevant information from the raw data. In this work, we used feature engineering to extract information from the NMR T_2_-relaxation distribution. The complete FE model derivation procedure is illustrated in Fig. 3.

In petrophysics, the T_2_-relaxation is regularly analyzed by geoscientists to determine fluid saturations in reservoirs, differentiate between different types of fluids, study the distribution of pore size, and evaluate physicochemical properties of fluids. However, depending on the task, some parts of the T_2_ distribution may have more relevance than others. In the context of studying the water content in oil-sands by NMR, we use feature engineering to reduce the amount of unnecessary information while preserving the data carrying the most information about the water in samples. A time-domain distribution of the T_2_-relaxation was obtained by processing the spin-echo signal decay using a mathematical inversion. As the T_2_ distribution has a form of a continuous function, the discretization was performed for data binning which simplifies the input of data into the machine learning model. After the discretization, the T_2_ data was presented as a frequency distribution by 52 bins, with each bin corresponding to a particular T_2i_ relaxation time in milliseconds. To limit the number of inputs, we defined five new NMR T_2_ features.

As the T_2_ distribution of relaxation times is represented on the semi-logarithmic scale, the standard parameter for representing the average T_2_ relaxation is T_2_ logarithmic mean (T_2lm_):

6 $$T_{2lm} = \exp \left[ {\sum \frac{{A_{i} }}{A} \cdot \ln \left( {T_{2i} } \right)} \right]$$

where A_i_ is an amplitude at the corresponding T_2i_ bin, and A is a total NMR amplitude. Empirical evidence shows the strong relationship between viscosity of fluids and T_2lm_, implying that in a water–oil system where distribution tends to be multimodal due to their different relaxation properties, the T_2lm_ provides a better measure central tendency favoring both fast and slow relaxing parts of the distribution.

To account for the variation in T_2_ distribution (i.e. narrow vs. wide peaks), the T_2_ standard deviation was defined as:

7 $$T_{2std} = \sqrt {\frac{{\sum (A_{i} - \mu )}}{N}}$$

where *μ* is the T_2_ distribution mean, and *N* is the number of the T_2_ bins.

The T_2p_ was defined as a location of a maximum value (peak) of the T_2_ amplitude on T_2i_ axis. This parameter is used in the petrophysical practice for the separation of bound and producible fluids and fluid typing, since T_2p_ gives an indication of whether the largest amplitude portion of the signal corresponds to low or high T_2_ values.

8 $$T_{2p} = \max \left( {f\left( {T_{{2_{1} }} } \right), \ldots ,f\left( {T_{{2_{n} }} } \right)} \right)$$

Intelligent algorithms like XGBoost have gained popularity due to their ability to generalize complex data dependencies in large datasets and achieve state-of-the-art forecasting results. However, a small dataset is used in this study, where the overlapping of water and oil T_2_ signals are likely to remain hidden or poorly represented. So, instead of allowing the algorithm to search through the whole NMR T_2_ distribution, we can ‘show’ it where to look for the patterns and changes in the amplitude. One of the important parts of the T_2_ distribution in sandstones is the empirical clay-bound water T_2_ cutoff located at 3 ms, which presents the boundary between capillary bound and clay-bound fluids in a water-saturated core^21,22^. In order to capture the possible T_2_ response of clay-bound water, and monitor its signal variation with different training samples, we defined a T_2_ bound fluid (T_2bf_) interval as:

9 $$T_{2bf} = \sum\nolimits_{{0.1\left( {ms} \right)}}^{{3.0\left( {ms} \right)}} {A_{i} }$$

However, this parameter cannot be used on its own to describe the changes in water content, since the oil signal may also be located in the relevant interval. The true T_2_ cutoff value in petrophysical practice is usually determined by performing lab tests on the saturated core samples (i.e. centrifuging), and even then the use of a fixed or averaged T_2_ cutoff value leads to the erroneous prediction of producible fluids. Instead, we attempt to obtain insights about the true T_2_ cutoffs using a feature extraction technique called Mutual Information (MI) regression, based on the information entropy between variables. In classical regression analysis, statistical tests like F-test are carried out to study the degree of the linear association or continuous analysis of covariance (CANOVA) for the non-linear association between variables. However, mutual information is not ‘concerned’ whether the variables have apparent linear correlation or covariance of zero, and they may still be stochastically dependent. This is the case in studying the changes in the conditional probability of one variable when another is modified^23^. In other words, by using MI regression, one can measure the level of association of the specific parts of T_2_ distribution with the target output (i.e. water content by Dean-Stark), regardless of their correlation or covariance. The score is measured in natural units of information or ‘nats’ which are based on natural logarithms and powers of *e*. The MI regression was performed on the training set using a Python library sklearn.feature_selection class mutual_info_regression.

**Figure 4:**
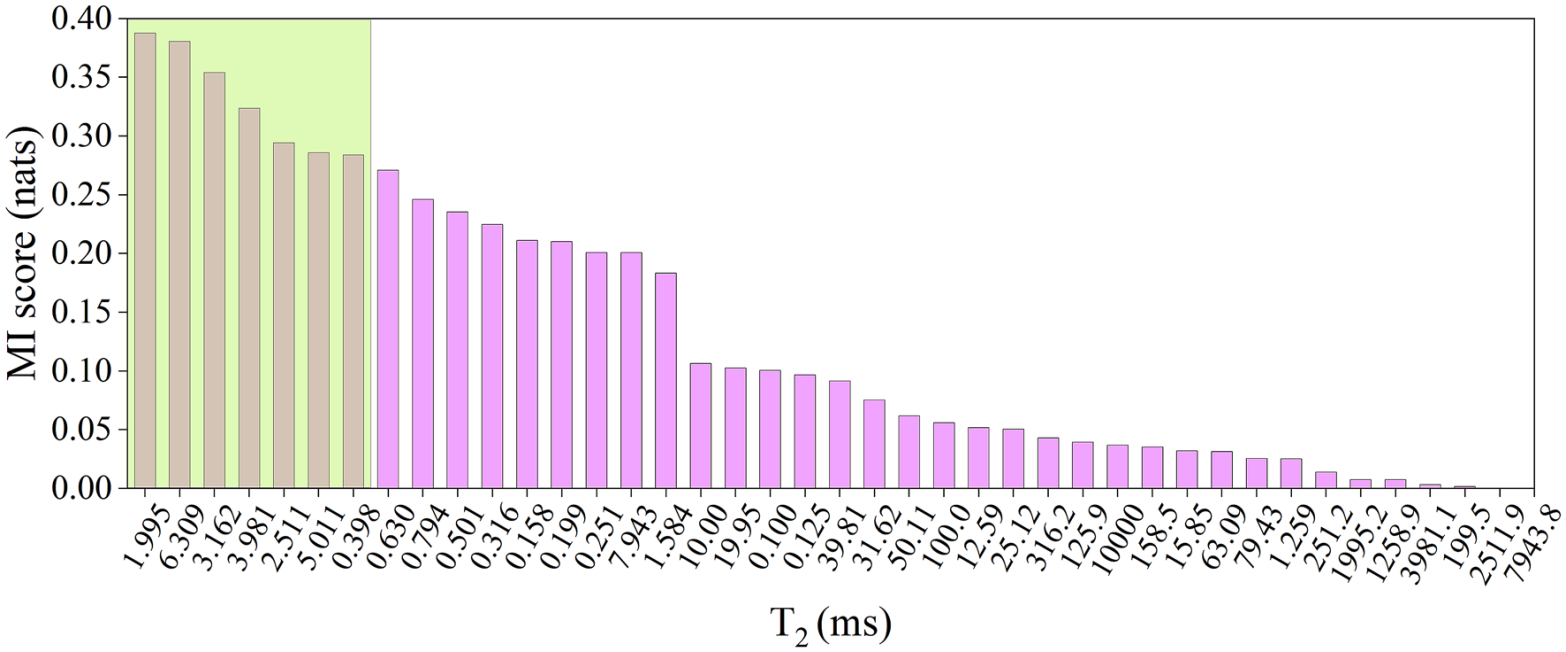
Results of the mutual information regression applied to the training set T_2_ distributions of the oil-sand samples relative to the Dean-Stark water content (DS-w). The shaded area presents the continuous cluster of T_2_ responses with a strong mutual association with DS-w, which were used for the calculation of the T_2_ cutoff range parameter – T_2cr_.

Figure 4 shows the relative mutual information scores of T_2_ responses, where higher values indicate a stronger association with water content by Dean-Stark. For this dataset, the responses from 1.99 to 6.30 ms have the highest association with the water signal and form a continuous cluster between 10^0^ and 10^1^ decades along the T_2_ semi-log scale, suggesting that most of the theoretical T_2_ cutoff values lie in this interval. Therefore, the T_2_ cutoff range (T_2cr_), was defined as:

10 $$T_{2cr} = \sum\nolimits_{{1.99 \left( {ms} \right)}}^{{6.30\left( {ms} \right)}} {A_{i} }$$

As previously mentioned, the T_2_ surface relaxation and diffusive-coupling play an important role in the identification of clay-bound water, which causes the overlapping of the water and oil signals. Unfortunately, for the determination of their contribution, a sample recovery for the subsequent lab experiments is required. However, a common practice in well-logging is to combine NMR and bulk density logs to improve interpretation. Therefore, the bulk density was used as an additional parameter which we postulate is associated with T_2_ surface relaxation and diffusive-coupling.

**Figure 5:**
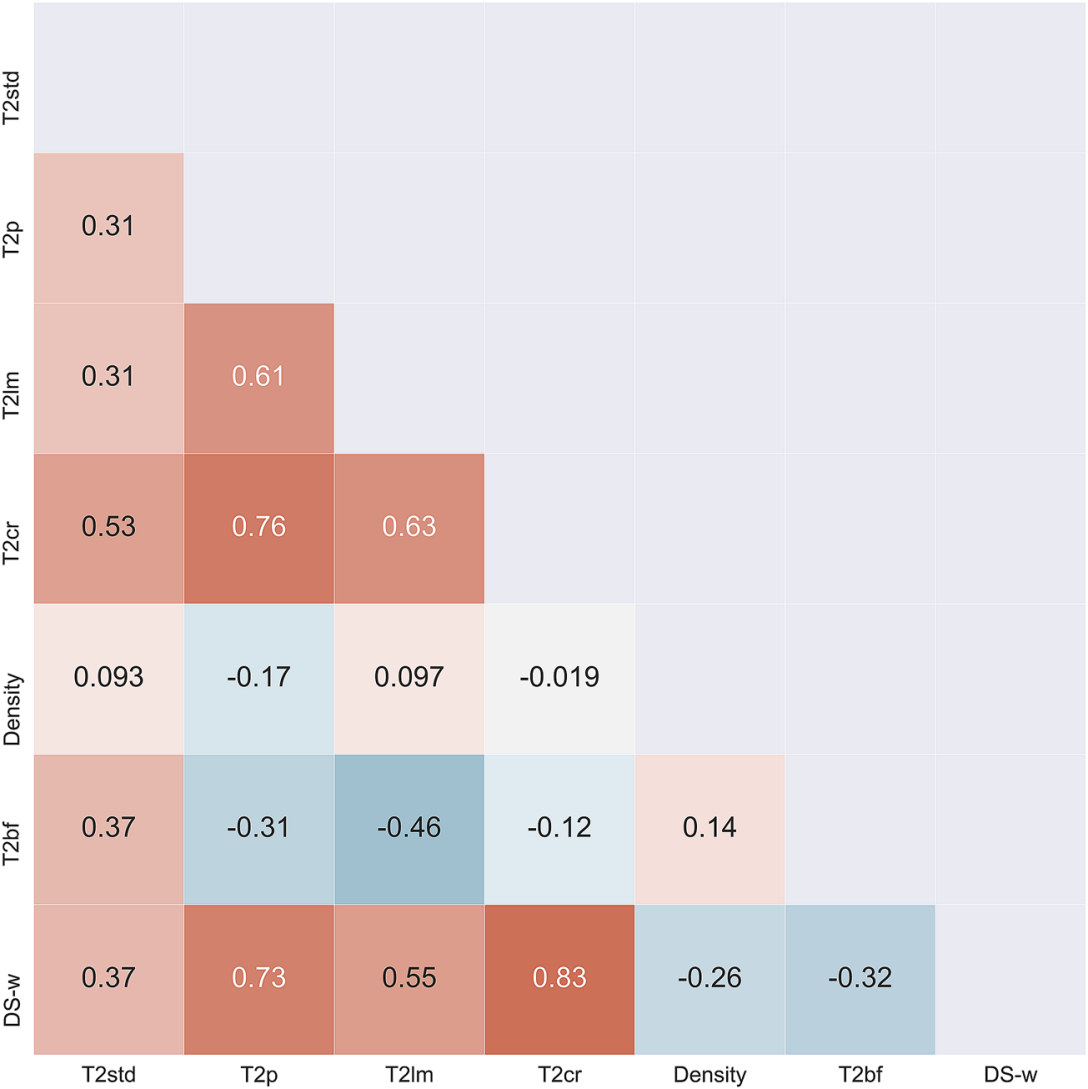
The diagonal correlation matrix showing the amount of linear dependence between six input features with Dean-Stark water content (DS-w). Scores represent the Pearson’s correlation coefficient and are color coded (heatmap).

**Figure 6:**
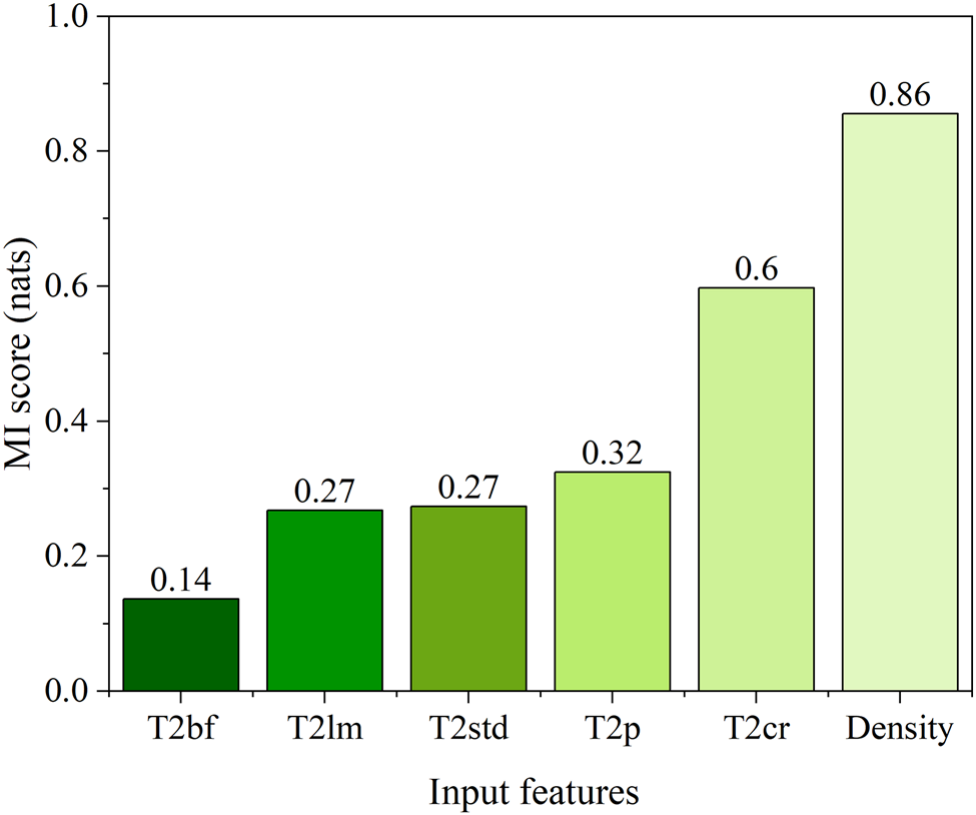
Mutual information regression scores for five NMR parameters and bulk density (input features) relative to the Dean-Stark water content (DS-w).

The correlation matrix (Fig. 5) shows the amount of linear dependence between the input features and target output. According to the Pearson score, T_2_ cutoff range and T_2_ peak and T_2_ logarithmic mean features exhibit the strongest positive correlation with the water content by Dean-Stark (DS-w). The T_2_ standard deviation shows a moderate degree of positive correlation, while T_2_ bound fluid and density features show moderate to low negative correlation with DS-w. Interestingly, when compared with mutual information scores from Fig. 5, it can be observed that all features are ranked by score accordingly to Pearson’s scores except for density which has the highest MI score (0.86 nats), indicating its strong stochastic (nonlinear) dependence with DS-w, thus justifying integration of density measurements into the model. Therefore, the XGB-FE model was developed using the six features presented in Table 2 (Fig. 6).

**Table 2:** Descriptive statistics of six input features used for XGB-FE model development.

Statistic	T_2std_ (a.u.)	T_2p_ (ms)	T_2lm_ (ms)	T_2cr_ (a.u.)	T_2bf_ (a.u.)	ρ (kg/m^3^)
Count	164.000	164.00	164.00	164.00	164.00	164,00
Mean	0.016	11.57	1.84	0.18	0.31	1626
Std	0.005	4.54	1.22	0.13	0.10	80
Min	0.006	1.00	0.32	0.00	0.10	1442
25%	0.013	8.00	0.90	0.07	0.23	1581
50%	0.016	13.00	1.61	0.18	0.30	1634
75%	0.019	15.25	2.49	0.29	0.38	1677
Max	0.032	20.00	8.59	0.50	0.54	1842

### XGBoost model based on the full T_2_ relaxation distribution (XGB-FS)

**Figure 7:**
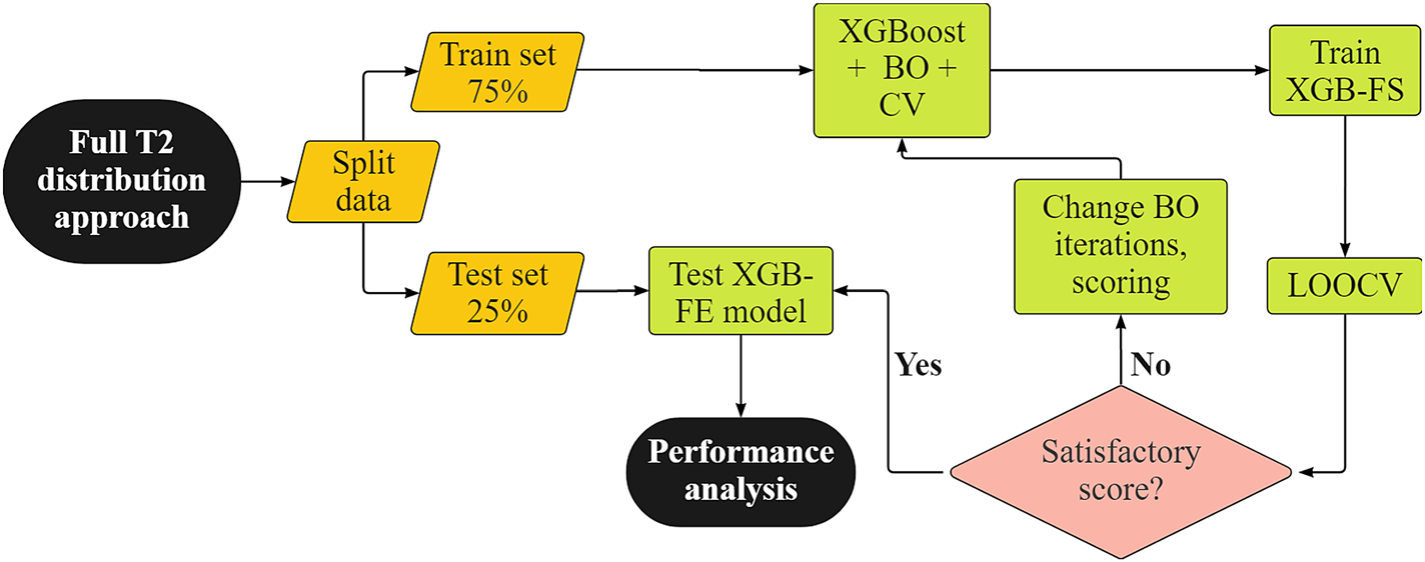
Flowchart for XGB-FS model development.

The second modeling approach facilitates the complete sample T_2_ distribution. There are two main incentives for this approach. First, the T_2_ relaxation distribution contains a large amount of information about the fluids residing in the pore space, indicating that the use of a single or even a few features to characterize the whole distribution, may lead to the significant information loss, and therefore to poor model forecasting performance^24^. By using the entire T_2_ distribution, variations such as changes in slope or local minima can implicitly be used to help separate oil and water signals. Secondly, predictions generated by the full-T_2_ distribution model provide a good baseline for comparison with the feature engineering approach and conventional deconvolution approach. Therefore, the input features were arranged as *X* = [*A*_1_, *A*_2_, *A*_3_, …, *A*_52_, *ρ*_*i*_] where *A*_*i*_ is the *i*-th column vector of the amplitudes at the corresponding *T*_*2i*_ bin, and *ρ*_*i*_ is a column vector of density measurements. The water content by Dean-Stark (DS-w) was arranged as *Y* = [*DS-w*_1_, *DS-w*_2_,…, *DS-w*_*n*_] thus defining the dataset as $\{\left({X}_{i},{Y}_{i}\right){\}}_{i=1}^{n}$ where $n$ is the number of oil-sands samples. The complete XGB-FS model derivation procedure is illustrated in Fig. 7.

### Model optimization

The XGBoost algorithm contains many hyperparameters which enable fine model tuning. From the standpoint of statistical learning, the tuning usually involves the use of iterative algorithms which search for a suitable combination of hyperparameters in real-valued parameter space, relative to the specified measure of model forecasting performance (e.g., mean squared error). However, as the number of parameters grows, the optimization becomes computationally expensive due to the combinatorial explosion, making the manual optimization or exhaustive grid searching techniques inefficient. In contrast, Bayesian Optimization (BO) sets a probabilistic approach where each successive combination of hyperparameters is selected based on the information obtained in the previous optimization step, thus avoiding the redundant calculations for unlikely parameter combinations and reducing the number of required iterations to reach the global minimum of the objective function.

The BO was performed in Python using scikit-optimize package class skopt.BayesSearchCV. The hyperparameters and their optimal values are presented in Table 3.

**Table 3:** Results of Bayesian Optimization with fivefold cross-validation, for XGB-FS and XGB-FE models.

XGBoost hyperparameters	Search range	XGB-FS optimal	XGB-FE optimal
n_estimators	[50–1000]	[650]	[300]
learning_rate	[0.004–0.1]	[0.008]	[0.053]
subsample	[0.7–1.0]	[0.7]	[0.6]
max_depth	[6–12]	[8]	[7]
objective	[‘squared_error’, ‘pseudo_huber’]	[‘pseudo_huber’]	[‘squared_error’]
grow_policy	[‘depthwise’, ‘lossguide’]	[‘lossguide’]	[‘lossguide’]
booster	[‘gbtree’, ‘dart’]	[‘gbtree’]	[‘gbtree’]

### Performance metrics and model validation

The forecasting performance of the models was evaluated using three performance metrics including coefficient of determination (R^2^), root mean squared error (RMSE), and mean absolute error (MAE). The R^2^ is the positively oriented metric used in regression for representing the amount of model variance, and how well the model predictions generalize the observations. However, R^2^ alone does not provide information on prediction errors. The RMSE is another regularly employed error metric, used alongside R^2^, but under the assumption that residuals follow the normal distribution^25^. As a result of the heavy penalization of larger residuals, the RMSE is a convenient metric for revealing the differences in performance between multiple models with normally distributed residuals. At the same time, large residuals can cause the inflation of the RMSE score, which is why MAE can be used for additional evaluation. The MAE measures the mean magnitude of model prediction errors, but in contrast to RMSE, the errors are not squared. Therefore RMSE scores are always greater or equal to MAE scores. These two metrics can be used together to estimate the variation in errors, where RMSE = MAE indicates no variation in the magnitude of prediction residuals. Note that both RMSE and MAE are negatively oriented scores expressed in %DS-w.

11 $${\text{R}}_{2} = 1 - \frac{{\mathop \sum \nolimits_{{{\text{i}} = 1}}^{{\text{n}}} ({\text{y}}_{{\text{i}}} - {\hat{\text{y}}}_{{\text{i}}} )^{2} }}{{\mathop \sum \nolimits_{{{\text{i}} = 1}}^{n} ({\text{y}}_{{\text{i}}} - {\overline{\text{y}}} )^{2} }}$$

12 $${\text{RMSE}} = \sqrt {\frac{1}{{\text{n}}} \mathop \sum \limits_{{{\text{i}} = 1}}^{{\text{n}}} ({\text{y}}_{i} - {\hat{\text{y}}}_{{\text{i}}} )^{2} }$$

13 $${\text{MAE}} = \frac{1}{{\text{n}}}\mathop \sum \limits_{{{\text{i}} = 1}}^{{\text{n}}} \left| {{\text{y}}_{i} - {\hat{\text{y}}}_{i} } \right|$$

where ${y}_{i}$ is predicted %DS-w, ${\widehat{y}}_{i}$ is observed %DS-w, $\overline{y }$ the sample mean, and *n* presents the number of samples.

The further model performance evaluation and validation were performed using leave-one-out cross-validation (LOOCV) due to its convenience for use on small datasets (Fig. 10). Cross-validation is a resampling method in which the sample subsets are drawn repeatedly from the training set, followed by model refitting for each subset, thus providing information on model fitting variability. In LOOCV, the samples are drawn for one observation at a time, while the rest of the data is used for model training. Therefore, this process has a number of iterations equal to the number of samples, making it computationally expensive for large datasets.

In addition to LOOCV, the permutation tests were conducted for assessing the significance of fivefold cross-validated model prediction scores with 150 random permutations. This enabled the evaluation of the statistical significance of model predictions and their inputs by a permutation test *P*-value.

## Results

**Figure 8:**
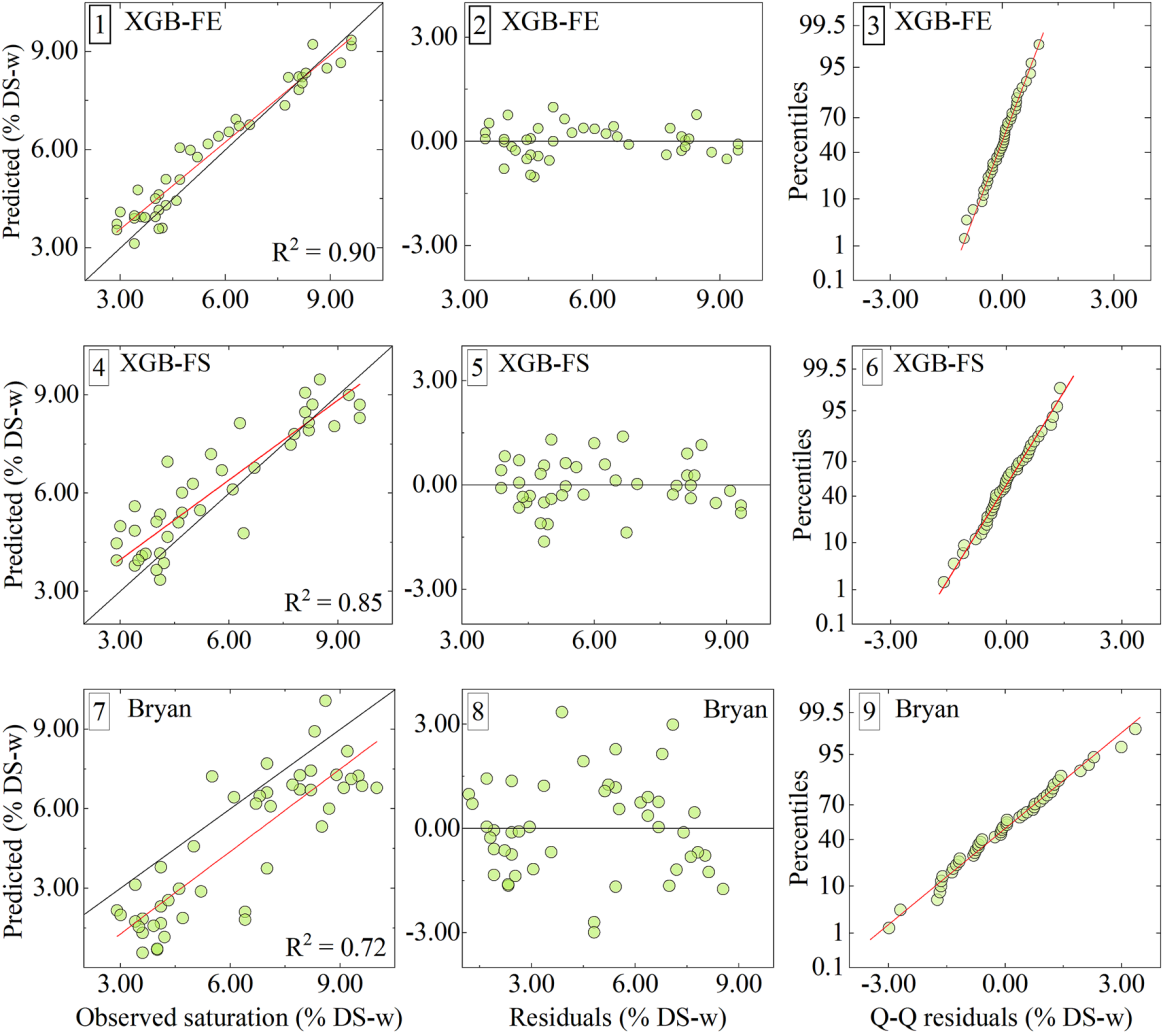
Evaluation of XGB-FE, XGB-FS and Bryan et al.^7^, performance by cross-plots between the model predictions and observed saturation in %DS-w (**1**, **4**, **7**), distribution of regular residuals (**2**, **5**, **8**), and quantile–quantile plots for comparing distributions between predictions and observations and evaluating normality of residuals (**3**, **6**, **9**).

**Figure 9:**
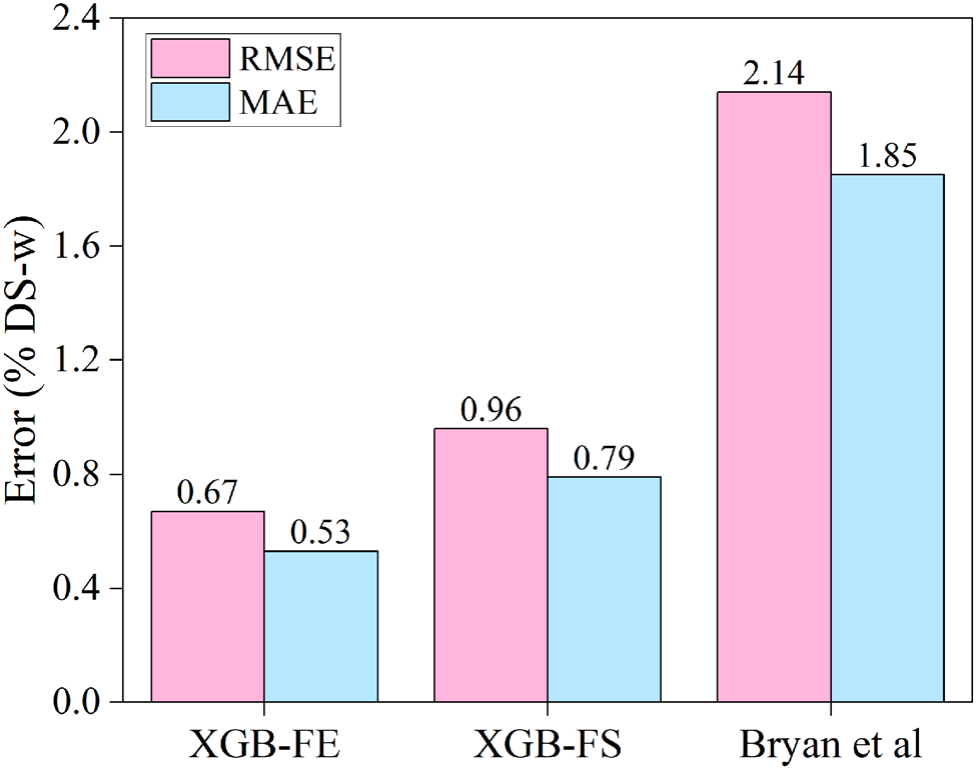
Comparison of RMSE and MAE test prediction scores for the three models (*‘random_state* = *2’*).

**Figure 10:**
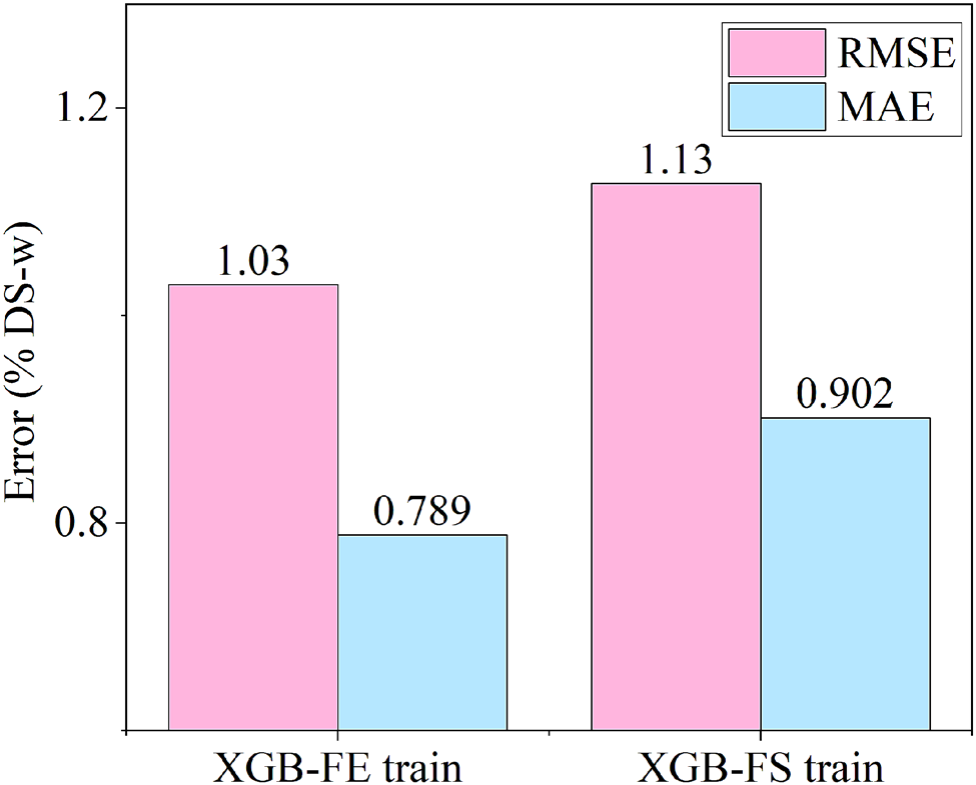
Leave-one-out cross-validation (LOOCV) scores for XGB-FS and XGB-FE machine learning models for the training set with fixed random split seed *‘random_state* = *2’.* Note y-axis was truncated for convenience.

In this section, the performance of three models is presented including the XGB-FE model based on the XGBoost algorithm with feature engineering, the XGB-FS model based on the XGBoost algorithm using the whole sample T_2_ distribution, and a peak deconvolution approach (Bryan et al^7^). To assess the model performance in more detail, residual plots (Fig. 82,5,8) and quantile–quantile plots (Fig. 83,6,9) are used for the analysis of the residual normality, and model variance and bias. All results are summarized in Figs. 8, 9 and 10.

Analysis and comparison of error statistics, cross-plots, and distribution of residuals indicate that the XGB-FE model achieves the highest accuracy and generalization ability in the prediction of water content in oil-sand samples. Figure 8-1 shows that apart from slight overprediction in the 3–5% DS-w range, all XGB-FE predictions spread along the x = y line, with low variance, achieving the highest R^2^ score in the study (R^2^ = 0.90). Figure 8-2 shows the constant low variance of the residuals, indicating that the model inputs capture variation in the data properly. Finally, the Q-Q plot (Fig. 8-3) confirms the residual normality and thereby the underlying assumption that XGB-FE model residuals follow the normal distribution (low bias, low variance). Finally, the XGB-FE model achieves 1.5–3 times lower RMSE and MAE scores compared to the XGB-FS and Bryan et al.^7^ models indicating the best generalization ability of the three.

As for the XGB-FS model predictions, Figs. 8-4,5,9 show a similar residual distribution to XGB-FE (normality and bias). The residual variance however is increased but constant, therefore achieving a somewhat lower R^2^ score (R^2^ = 0.85), and 1.5 times higher RMSE and MAE compared to XGB-FE. From Fig. 8-7, it can be observed that the Bryan et al.^7^ model generally tends to underpredict the water content in samples. In addition, Fig. 8-8,9 show inflated but constant variance in the distribution of residuals, while residual normality still holds with some local perturbing. As a result, Bryan et al.^7^ model RMSE and MAE scores are the highest of the three (Fig. 9).

## Discussion

The two machine learning models in this study were designed to test two principal hypotheses. First, to confirm that integration of density measurements into the machine learning models can help to separate the contribution from overlapping oil and water signals. Second, to show that the derivation of new LF-NMR T_2_ features can improve the generalization ability of the machine learning model to a degree that can enable the accurate forecasting of water content by LF-NMR in oil wells (in-situ).

Bulk density measurements are regularly used together with LF-NMR measurements in petrophysical practice to improve the interpretation of well logs^4,26^. LF-NMR measures the response of the fluids in the rock pore space, therefore carrying information about the fluids and pore size distribution of the rock. On the other hand, density logging equipment measures the response of the solids (rock matrix), together with fluids. The two are related in terms of T_2_ surface relaxation which depends on the rock pore to surface ratio with the fluid volume, as well as with diffusive-coupling effect. This dependence can be also observed from the prediction test scores of the XGB-FS and XGB-FE models with and without bulk density as one of the model inputs. Prediction scores from Tables 4 and 5 indicate that models achieve better scores with the integration of bulk density, therefore confirming the relationship between Dean-Stark water content and density discovered by mutual information regression.

**Table 4:** Comparison of XGB-FS model performance with and without *bulk density* parameter.

XGB-FS		
Statistic	wo/Density	w/Density
RMSE (%DS-w)	1.08	0.96
MAE (%DS-w)	0.86	0.79
R^2^	0.72	0.85

**Table 5:** Comparison of XGB-FE model performance with and without *bulk density* parameter.

XGB-FE		
Statistic	wo/Density	w/Density
RMSE (%DS-w)	0.91	0.67
MAE (%DS-w)	0.73	0.53
R^2^	0.81	0.90

To affirm the second hypothesis: when XGB-FS and XGB-FE are compared (Fig. 8), it can be observed that XGB-FE achieves better performance, especially in terms of prediction variance. The XGB-FE variance reduction supports two premises. First, the engineered features properly capture all the relevant information from the T_2_ distribution, indicating negligible information loss. Secondly, in the feature engineering case, the XGBoost algorithm generalized the variability in the data with the output more effectively, suggesting that for smaller datasets the appropriate feature engineering enables the XGBoost algorithm to discover dependencies within the data more effectively than for a large number of raw information (53 features in case of XGB-FS). In other words, the new features contain all the relevant parts of the T_2_ distribution compressed into a few values, which ultimately reduces the XGB-FE model complexity and therefore enables better generalization of the relationship between inputs and a target variable (DS-Sw). According to Fig. 6, along with a bulk density (MI = 0.86 nats), the T_2_ cutoff range feature ranks second by MI score (0.60 nats), indicating the variability of the sum of T_2_ responses between 1.99 and 6.30 ms has a strong relationship with water signal. The location of the T_2_ peak (T_2p_), T_2_ standard deviation of the spectrum (T_2std_), and T_2_ logarithmic mean (T_2lm_) achieve similar MI scores (0.27 and 0.30 nats, respectively) signifying that these features alone do not capture enough information about the water content. Finally, the sum of T_2_ responses representative of the empirical clay-bound water part of the T_2_ distribution (0.1–3.0 ms) shows the least association with the target (DS-w). Although these features alone cannot explain variance in data effectively, their mutual interaction can improve it. Since MI does not consider this mutual interaction between features relative to the target output, the correlation matrix can be used. For instance, Fig. 3 shows that the T_2cr_ versus T_2p_ and T_2cr_ versus T_2lm_ have a strong positive correlation (0.76 and 0.63 respectively), while T_2bf_ versusT_2lm_ have a moderate negative correlation. These interactions are likely to be generalized in the XGB-FE model training process, thus explaining its improved performance. Furthermore, the permutation test score of XGB-FE using 150 permutations generated a *P*-value of 0.001, compared to the XGB-FS P-value of 0.007. In both cases the *P*-value is well below 0.05, showing a very low likelihood of obtaining such model performance purely by chance.

As for the deconvolution approach (Bryan et al.^7^), the main challenge lies in the separation of overlapping fluid contributions in T_2_ distribution. Even under the assumption that T_2_ cutoff and deconvolution are performed such that a precise distinction between fluid signals is possible, the issue of how to associate the amplitudes with respect to mass persists. For the given dataset, this approach leads to underprediction of water content, indicating that the oil and water signals are not sufficiently separated. The machine learning-based approach is more robust because it removes the necessity to manually identify peak separation and the errors associated with visually separating oil and water signals, especially in the case of NMR measurements acquired at low SNR.

It is important to point out the limitations of these models which are related to reservoir lithology (1) and SNR of the measurements (2):

1. The models presented in this study were derived for the oil-sands reservoir, which is why their application is limited only to similar reservoir types. However, the presented approach can be extended for use in other types of oil reservoirs, under the assumption that a sufficiently large amount of observations is available.
2. The SNR achieved by the benchtop LF-NMR relaxometers can be up to 30 times higher than the SNR values obtained using well-logging tools. In this study, the NMR signal-to-noise ratio was on average 20, which can be still considered high relative to the logging tools where the SNR of 3–5 is considered satisfactory^27^. Although the recent research demonstrated that XGBoost algorithm is sufficiently robust even with noisy data^28^, an additional validation using the data obtained by the LF-NMR logging tools would be desirable. It is worth noting that, in lower SNR samples, the deconvolution approach will be even more challenging, and the value of using just the general properties of the T_2_ distribution and XGBoost may be even further enhanced.

As a follow-up study, the procedures for NMR measurements with a controlled saturation and desaturation of samples, similar to those reported in recent literature^10^, would enable deeper sensitivity analysis of the features derived in this work and further improvement of the XGB-FE model. In such a setup, the Dean-Stark measurements could be replaced by the more cost and time-effective mass-volume measurements, ultimately allowing the collection of a larger database at which point the application of artificial neural networks (ANNs) would be possible.

It is also worth noting that logging equipment configuration can be substantially different from desktop NMR relaxometers, which may cause inconsistencies between NMR T_2_ distributions obtained in the lab and the field. This can cause the variable performance of proposed NMR data-driven model, which is why the parameters of the NMR logging device, such as TW, TE and number of trains, should be relatively consistent to the values reported in this study.

## Conclusions

This study presents the approach which integrates extreme gradient boosting with LF-NMR measurements and bulk density data for the water saturation determination in oil-sands. Two models were developed using full NMR T_2_ distribution (XGB-FS), and feature engineering (XGB-FE). It is concluded that;

- Feature engineering can be effectively used to extract vital information from NMR T_2_ distribution, using domain knowledge and mutual information regression.
- The integration of bulk density data as a model input notably improves the XGB-FS and XGB-FE forecasting performance.
- XGB-FE achieved RMSE = 0.67%, MAE = 0.53% and R^2^ = 0.90 in predicting relative water content by Dean-Stark, a substantial improvement compared to deconvolution method.

These results suggest that the XGB-FE model can be extended for the improved *in-situ* water saturation determination.

## Data availibility

Correspondence and requests for materials should be addressed to S.M.

## Abbreviations

LF-NMR: Low-field nuclear magnetic resonance
OOIP: Original oil in place
EOR: Enhanced oil recovery
TE: Echo spacing
RMSE: Root mean square error
MAE: Mean absolute error
XGB: Extreme gradient boosting
FE: Feature engineering
FS: Full spectrum
XGB-FE: Extreme gradient boosting model based on feature engineering
XGB-FS: Extreme gradient boosting model based on full T_2_ distribution (spectrum)
DS: Dean-Stark
DS-w: Water content by Dean-Stark
L1: Lasso regression
L2: Ridge regression
CPMG: Carr-Purcell-Meiboom-Gill pulse sequence
SNR: Signal-to-noise ratio
CANOVA: Continuous analysis of covariance
MI: Mutual information
BO: Bayesian optimization
LOOCV: Leave-one-out cross-validation
X-ray CT: X-ray computed tomography
BSS: Blind-source signal separation
ANNs: Artificial neural networks
